# Digital Entrepreneurship: What is New if Anything?

**DOI:** 10.1007/s12599-021-00741-9

**Published:** 2022-02-03

**Authors:** Dennis M. Steininger, M. Kathryn Brohman, Jörn H. Block

**Affiliations:** 1 Faculty of Business Studies and Economics, Technical University of Kaiserslautern, Kaiserslautern, Germany; 2 Smith School of Business, Queen’s University, Kingston, Canada; 3 Faculty of Management, Trier University, Trier, Germany; 4 Erasmus School of Economics and Erasmus Institute of Management (ERIM), Erasmus University Rotterdam, Rotterdam, The Netherlands; 5 Witten Institute for Family Business (WIFU), Witten/Herdecke University, Witten, Germany

## Introduction

Opportunity identification and exploitation are at the center of entrepreneurial activities (Shane and Venkataraman 2000; Short et al. 2010; Zahra and Dess 2001). Such opportunities can emerge through new digital technologies (i.e., the combination of information, computing, communication, and connectivity technologies, Bharadwaj et al. 2013) as well as through the change and disturbances that are brought about by these technologies in economy and society (Keen and Williams 2013).

Our motivation in producing this Special Issue was to study whether established assumptions underpinning entrepreneurship theories still hold in the digital age, i.e., during the emergence and impacts of digital technology and related opportunities (Berger et al. 2021; Block et al. 2020; Steininger 2019; von Briel et al. 2021). Inspired by the focus of *Business & Information Systems Engineering* to examine problems related to the development, implementation and management of information systems, we propose to contextualize principles of digitalization to the entrepreneurship field. As guest editors (Dennis Steininger, Kathryn Brohman, and Joern Block) bring different perspectives, we aim to clarify the study of digital entrepreneurship by identifying what has changed and what remains the same.

Before discussing underlying assumptions and summarizing the articles chosen for publication in this Special Issue, we would like to thank all authors that responded to our call for papers. Reviews were completed by scholars that conduct research at the intersection between information systems and entrepreneurship. We identified cross-disciplinary review panels for each paper to ensure our evaluation went beyond one of the involved communities. After a first review, we invited the authors of five papers to revise and resubmit their manuscripts. Guest editors worked closely with the authors to offer suggestions and provide constructive feedback. In the end, all articles that were included in the Special Issue span a wide range of the digital entrepreneurship phenomenon including changes to agency relationships, evolving digital capabilities, public policy, and the impact of artificial intelligence (AI).

## Theoretical Implications of Digital Entrepreneurship

In this section, we challenge assumptions by drawing out some key themes related to the potential impact of digitalization on traditional entrepreneurship, highlighting areas in which existing theory may or may not suffice. First, it is important to discuss the evolution of different technology characteristics and how changes in technology have influenced the study of entrepreneurship. Since uncovering the advantages of Internet technologies in the 1990s, to exploring the role of open source and social media in the 2010s, and now examining the influence of big data and blockchain, the key insight for researchers is that characteristics of digital technologies differ from those inherent in traditional IT (Nambisan 2017; von Briel et al. 2021; Yoo et al. 2010). Digital technologies (i.e., combinations of technologies related to social, mobile, analytics, cloud, Internet of Things (IoT), and platforms, (Vial 2019)) are unique in that re-programmability, homogeneity of data, and self-referential nature yield a different set of entrepreneurial outcomes as they are easier to combine to create innovation (i.e., convergence) and enable unprompted change (i.e., generativity) (Lyytinen et al. 2016; Yoo et al. 2010). The uniqueness of digital technologies also calls attention to the difference between traditional IT capabilities and digital capabilities with the latter being defined as more appropriate for leveraging technology resources for innovation purposes (Wiesböck et al. 2020). However, new digital technologies typically do not create economic value per se, related emerging opportunities need to be identified and pursued for value creation. At the same time, the process of opportunity recognition and exploitation can thereby be influenced by digital technologies itself, as one of the papers in this Special Issue elaborates (Kreuzer et al. 2022). This relates to the discussion about the self-referential nature of digital innovation (Yoo et al. 2010). One of the main tools for entrepreneurs to pursue opportunities by creating and capturing value from new technologies is the business model concept. It allows entrepreneurs to specify required activities involved in pursuing an opportunity, define the activities’ enablement via technology, and link them with an overall value creation and capturing logic (Osterwalder et al. 2005; Zott et al. 2011; Zott and Amit 2010). Depending on their characteristics and deployment by entrepreneurs, various forms of technologies can play differing roles in creating diverse types of new ventures and business models (Steininger 2019). Leveraging the different dimensions of new ventures’ business models provided by Al-Debei and Avison (2010), we contextualize principles of digitalization in the entrepreneurship field by discussing some of the changes induced by technology below.

### Value Proposition and Product

New technologies fundamentally enable the resolution of not-yet-addressed customer pain points or unidentified needs. For example, the application of sensors, digital video, large road regulation data sets (enabled by the homogenization of data (Yoo et al. 2010)), and AI can bring autonomous vehicles to the roads, reduce drivers' pain during heavy traffic, while at the same time reduce traffic fatalities caused by large numbers of accidents. The development of such autonomous agents is a good example of how digital technology can generate new value through different types of business models (e.g., pay-per-use for physical products) and novel user interactions. The topic of (disruptive) entrepreneurship emerges when new business models eradicate existing business models that rely on in-person interaction such as automobile service appointments, taxi services, police enforcement of speeding fines, and writing tickets for parking infractions. The rapid emergence of start-ups that use digital technologies such as AI to alter workforce-intensive tasks is the topic of one of the papers of this Special Issue (Weber et al. 2022). Another pain point relates to how accelerated scaling and increased autonomy offered by digital technology are making customer relations more volatile. This calls attention to entrepreneurial activity related to digital servitization and the emergence of service bots, self-service interfaces and service ecosystems that may challenge established theories. Examples of this include the assumed liability of newness and smallness (Abatecola et al. 2012; Stinchcombe 1965) as digital entrepreneurship may reduce resource scarcity via technologies such as AI or crowdsourcing/crowdfunding and the use of stage theories that have informed the product development process for decades as we describe in more detail below.

The characteristics of digital entrepreneurship outlined above become even more critical for value creation due to the network externalities that are common for many new ventures in the digital arena. Users thereby become an integral part of the value proposition. This means that the more users (and data about users) an organization has, the more potential value their offers will generate. This introduces a new set of complexities for entrepreneurial ventures that are driven by a cold start problem and highlight the need to reach a critical mass of users and/or data to establish a strong resource base (Katz and Shapiro 1992). These inherent characteristics of digital entrepreneurship create tensions for start-ups with scarce resource endowments (i.e., liabilities of newness and smallness, Stinchcombe 1965). Moreover, these tensions provide incentives for start-ups to operate at the edge of what is legally permissible or exploit areas that have not yet been regulated. Take for example data collected and governed by companies like Google and Facebook or the rental of living space to short-term guests via AirBnB. Such activities allow new ventures to leverage outside resources for managing tensions of resource scarcity and network externalities but have, at the same time, put regulatory questions in the spotlight. Moreover, due to network externalities rendering such industries as winner-takes-all markets, early movers have often become dominant players with the possibility to build entire ecosystems (leveraging the convergence of digital technologies and their modular layered architecture, Lyytinen et al. 2016; Yoo et al. 2010) and thereby establishing new roles of entrepreneurial actors.

Platforms and two-sided markets are good examples of how digitalization leads to the emergence of new actors. Platforms as intermediaries change how start-ups interact with their suppliers as well as their customers. Equity and reward-based funding platforms, for example, have emerged as important players in entrepreneurial finance bringing together supply and demand sides to help start-ups raise money (Block et al. 2018). In some cases, established providers of entrepreneurial finance (e.g., banks, business angels, venture capital firms) adjust to this development and change their role becoming active players on these platforms as well. Whether (equity) crowdfunding and traditional forms of financing are complements or substitutes has received a lot of interest in the literature (e.g., Drover et al. 2015; Moedl 2021; Signori and Vismara 2018). The answer to this question is of high relevance and depends on the quality of the ventures that get funded and the openness of traditional entrepreneurial finance providers towards crowdfunding. The gig or sharing economy is another example where digital technology has had a strong impact on entrepreneurship (Burtch et al. 2018). Digital platforms such as Uber and 99designs give freelancers or solo-entrepreneurs the opportunity to offer their services to a wide range of customers or users and increase their potential reach and market size. The negative consequences include increased dependence on the platforms and increased competition with negative impact on margins and profitability (Ahsan 2020). As with the example of crowdfunding platforms, established providers of services need to create platforms themselves, push for regulation (see below), or become active on the platforms. The common thread in these examples is that digital technology has led to a change from a set of pre-defined actors (e.g., suppliers and users of a product or service) to a platform-based network or ecosystem that organizes transactions between an evolved set of different parties and/or user groups.

### Value Architecture and Network

It was mentioned earlier that new digital technologies do not create economic value per se, but fully digital business models (e.g., cloud services) in the narrow sense can leverage digital technologies for value creation, capture, and delivery (Steininger 2019). This creates changes in many parts of the value architecture and network dimensions of the business model. First, technological (i.e., digital) infrastructure within and across firms becomes crucial for digital entrepreneurs to create value. We thereby see a tendency that regions or countries with weaker digital infrastructure and standards become places that create less opportunities for digital entrepreneurs or at least make their road to success more troublesome (Tongia 2007). On a regional level, digitalization can lead to regional inequality (Haefner and Sternberg 2020). Relating this to the digital divide we can refer to the “digital access divide” where access for organizations or individuals to exploit digital opportunities is limited through missing or weak infrastructure (Wei et al. 2011). This can be worsened via missing or thwarting regulation and standard-setting, as mentioned in the interview of this Special Issue (Steininger 2022).

Second, and further following the idea of the digital divide, new capabilities (i.e., digital capability divide, Wei et al. 2011) are needed by digital entrepreneurs to successfully establish new digital business models. Agile and user-centric thinking enable “ever-in-the-making” products and new measures are needed to secure such offerings and ensure their constant availability, particularly for services delivered via the cloud (Lehmann and Recker 2022). Moreover, the ubiquity of digital products and services combined with lower propensity to pay strong salaries (due to scarce resources) on the one hand and scarce labor supply on the other, leads to higher demand for new forms of distributed work. This demand is heightened by the scarcity of digital experts, the COVID-19 pandemic, as well as the tendency of start-ups to recruit development teams in regions of the world where the supply of such experts is higher and wages are more affordable. As such, entrepreneurs depend on digital tools to streamline the work of team members spread across regions and this dispersed way of working requires different leadership capabilities of entrepreneurs to onboard employees and keep them updated and motivated (Petry 2018). One way that has become prominent during the last years is to approach this challenge by “working out loud” and “leading out loud” (Bartlett 2016; Stepper 2015) enabling more transparency of work and decisions in distributed teams.

Third, leveraging the multi-layered architecture of digital innovations (Yoo et al. 2010), the value creation and delivery have often become much more dependent on partners that contribute to a full user experience. For example, most digital start-ups rely on cloud infrastructure (e.g., Amazon’s AWS) as opposed to running their own data centers and thereby become very dependent on these providers. Moreover, when entrepreneurs enter a market by participating in an ecosystem (e.g., Google Play Store, Apple App Store), they must learn how to adhere to the governance rules and adopt business model configurations provided by the ecosystem. Particular challenges can arise when they are banned from further participation in the ecosystem (e.g., through changes in the governance) or threatened by ecosystem providers that introduce their own, competing technology or app. In this reign, ecosystem dominance can also shift competition and thereby hinder innovation, which makes regulation a very important, yet underdeveloped, aspect of digital entrepreneurship.

### Value Finance

Many digital new ventures struggle to find a viable revenue model due to the inherent characteristics of their value proposition. For example, Twitter struggled for over a decade to seek a sustainable way to monetize the users it amassed (Mangalindan 2010; Urstadt 2008). This struggle is particularly challenging when building business models in markets with network externalities due to the tension that exists between scarce resources (and therefore a need to monetize) and the critical mass of users that needs to be reached. Making a wrong decision in the early stages of such new ventures can create long-time path dependencies that leave a start-up struggling. Hence, it is critical to understand that pricing schemes have to be set very carefully in such contexts; specifically, start-ups need to either price below or extend/enrich the network value. If the pricing scheme is wrong, new ventures will experience a slow-down in user growth that can destroy long-term competitiveness. An example of a start-up that managed these tensions well is LinkedIn. On the one hand, they acquired several funding rounds to support network growth and provide free access to the large regular user base (i.e., pricing below network value). On the other hand, they introduced highly-priced memberships for recruiters that would gain much more value from using the network than regular users and in turn add value to the network by providing job offers. This allowed LinkedIn to speed up network growth while monetizing a specific customer segment as a primary source of income (Steininger et al. 2013).

Closely related to such questions of monetization are challenges of start-ups to raise funding. As seen with the example of LinkedIn, being able to raise enough capital over long time periods can be particularly crucial for digital entrepreneurs to grow a critical mass of users. However, digital means have enabled new ways of funding such as crowdfunding or Initial Coin Offerings (ICOs) that can also act as market tests (Block et al. 2021; Maier et al. 2021; Viotto da Cruz 2018). As such, digital technologies have shifted power and transparency in new venture funding. For example, in ICOs, founders can often pre-select investors based on their a priori inputs into the development of a project within private token sales. This can put start-ups in previously unprecedented position of strength. However, crowdfunding and ICOs can also provide overwhelming amounts of resources (i.e., overfunding) that leave start-ups struggling to manage sudden high inputs and demand, a phenomenon that was not known prior to the introduction of digital ways of funding new ventures (Bruckner et al. 2021). A new transparency introduced by the need of crowdfunding to openly and frequently communicate about current status and product development has put new ventures under pressure. They now need to find ways to use slim resources to engage more with the public and manage related issues such as online firestorms. Moreover, investors expect much more transparency of day-to-day business activities to be made available via digital channels (e.g., Slack) further increasing the workload of entrepreneurs to handle these demands (Steininger 2022).

### Value and the Development Lifecycle

From a non-digital perspective, the life cycle of start-ups from conception to stability remains relatively the same. Successful market entry still expects new ventures to evolve in some form of pre-launch, initial launch, early growth, scaling and establishment (Kazanjian and Drazin 1990; Srivastava and Shainesh 2015). However, the ambivalent properties of digital technology (Kallinikos et al. 2013) has changed the overall speed of this lifecycle and offers a refined set of entrepreneurial strategies to adapt to the changing business context. In terms of speed, digital technology’s inherent capacity to manage uncertainty drastically reduces the time and effort required to generate and evaluate ideas (Steininger and Gatzemeier 2019), develop and frame the opportunity, and prototype and launch a viable product or service (von Briel et al. 2018). Inherent in the speed is a fundamental change in assumptions that new ventures can enter the market with inherently unfinished products or services (McDonald and Eisenhardt 2020). Digitally enabled or fully digital products thereby tend to be “never-finished” or “always beta” versions. On the one hand, this allows positive effects such as over-the-air updates or bug-fixes for digitally-enabled physical products (e.g., Tesla often fixes bugs in their cars via over-the-air updates). On the other hand, it also poses new challenges to start-ups to enable continuous development and deployment of updates during runtime without outages (e.g., cloud software) as the authors of one of this Special Issue’s papers study (Lehmann and Recker 2022). Dropbox is a good example of this as their initial offering was an introductory video and sign-up function only. They leveraged technology to capture and evaluate customer feedback and used feedback to create new features and functionality beyond the early stages of the lifecycle and implement changes into a running service.

At the core of the refined set of strategies are new assumptions that emerge from the fact that properties inherent in digital technologies can change the form, function or purpose of how technology is used. Take for example the influence of social media fundamentally changing the way start-ups interact with potential customers (de Zubielqui and Jones 2020). For entrepreneurial ventures in non-technology based sectors, leveraging technology to speed up the lifecycle and alter strategies is definitely more novel and new. However, even for technology-based ventures, digital technologies continue to extend the realm of possibility as demonstrated by papers in this Special Issue.

Related and also strongly impacting product innovation processes, managing intellectual property (IP) is often rendered more complex and sometimes almost impossible for digital products or services (Miric et al. 2019). This depends strongly on a start-up’s country of origin and its legislation. Software patents, for example, are much more prevalent in the US than in Europe (Leifeld and Haunss 2012). Typical approaches related to patent-driven spin-offs in high-tech contexts thereby become much less important if not fully obsolete. This challenges several streams of innovation and entrepreneurship research that have applied the numbers and citations of patents as important proxies to measure (innovation) success and the value of high-tech firms (Harhoff et al. 2003).

A strong tension in product innovation that has gained more and more prominence during the last few years is the trade-offs between ethical and value-creating use of private user data. Specifically, adhering to strict privacy regulation on the one hand and creating a strong value proposition by enhancing the user experience on the other hand. Examples can be found in social media start-ups that rely on advertising business models that require the use of fine-grained data for targeting. There is clear evidence that the more control social media platforms give to their users (e.g., who can use and see their data), the more content the users will share in the network (Steininger 2016). These paradoxical tensions make product and service development for digital start-ups even more complex as they need to provide value to users and capture value for the firm while at the same time align with regulations of the target market.

### Value-Based Policy and Recognition

Policy makers consider entrepreneurship to be an important determinant of economic growth and development, hence the motivation to support entrepreneurship through public policy. However, the positive impact of entrepreneurship is not a sufficient justification. Many of the most successful start-ups responsible for innovation and growth are created without public support and taxpayer’s money (Shane 2009). Entrepreneurship policy needs a strong economic rationale based on market failure/imperfection or positive externalities (Acs et al. 2016). Digital entrepreneurship changes the rationales and functioning of entrepreneurship. As we describe below, regulatory work becomes particularly important as it strongly impacts the scope of action for entrepreneurs, opens up new opportunities, and shifts start-ups in a certain direction.

Entrepreneurship policy is concerned with market concentration and monopolistic behavior by incumbent firms that may limit market entry and can have negative effects on innovation and competition. This danger is increased with digital markets where technologies and inherent characteristics of platforms and platform ecosystems often enable exponential user and network (value) growth leading to lock-in situations for users and winner-takes-all markets. Hence, there is an increased need for entrepreneurship policy to create an equal playing field for start-ups versus tech giants such as Amazon or Google.

To make matters more difficult, platform monopolies can typically combine their data and services allowing another type of monopoly (i.e., data monopoly) that hinders further competition. Potential problems arise when only one venture can leverage these data via AI to improve user experience and value. This can start the “virtuous cycle of AI” (i.e., more and better data, leading to better product, leading to even more users) (Ng 2019) and manifest the monopolies (Gregory et al. 2021). Hence, (digital) entrepreneurship policy should not only focus on providing equal access to networks and their users, but also equal access to data generated by and through these networks. Overall, the vast potential of data collection enabled by new technologies (e.g., IoT, sensors) and platform business models has not only created opportunities for entrepreneurs, but also highlighted the importance of IT security and privacy as well as questions of data ownership and access. Regulators across different parts of the world have approached these types of challenges very differently. While winner-takes-all markets have seldomly been clearly identified and regulated as monopolies, privacy regulation has been the focus of several regulators. As the United States installed relatively liberal privacy protection (which enabled the upsurge of dominating new players of a data-driven economy), European countries adopted a much more restrictive approach with the General Data Protection Regulation (GDPR). Restrictive regulation prevented several types of data-driven business models in Europe; however, these restrictions also created new opportunities for start-ups targeting privacy sensitive customers. For example, with every privacy breach or data leak, concerned customers of US cloud services kept moving their data to firms that provided GDPR conform, encrypted cloud services hosted on European servers.

Next to entry barriers, entrepreneurship policy is also concerned with information imperfections arising from information asymmetries between start-ups and their resource providers. While digitalization has generally facilitated the provision of badly needed resources for start-ups through new intermediaries in the form of crowdfunding or digital labor platforms, it also creates challenges for entrepreneurs with digital products and services. As noted above, it is challenging to protect digital products and digital knowledge through intellectual property rights and other forms of protection such as secrecy. This can create a problem for digital start-ups to raise capital from banks and other traditional providers of corporate finance. Hence, there is an increased need for venture capital and other forms of entrepreneurial finance. This need is further strengthened by the winner-take-all logic that exists in most digital markets, where start-ups need to scale up fast. To what extent governmental venture capital (Brander et al. 2015) is needed or the provision of entrepreneurial finance should be supported through tax breaks (Keuschnigg and Nielsen 2003) or other forms of (in)direct subsidies is a question of high interest. In any case, many governments around the world have created specialized funds and various forms of tax subsidies to support (digital) entrepreneurship and innovation.

Finally, entrepreneurship in combination with innovation policy is concerned with positive externalities and knowledge spillovers from start-ups and their knowledge-based innovations. Knowledge, and to some extent also innovation, is a public good and spills over from one firm to another firm. Entrepreneurship policy aims to facilitate these spillovers to foster innovation and growth on the macro level. This implies that for digital products and services, the underlying code, data and algorithms should be made transparent and publicly available. In this way, digital entrepreneurship policy goes hand in hand with open source and open data policy.

## The Articles in the Special Issue

Moving now from the general trends of digital entrepreneurship research to the more specific studies chosen for the Special Issue (for an overview see Table 1), the articles draw from varied settings and phenomena and analyze how entrepreneurs are using digital technologies to bring changes in entrepreneurial processes, innovation, competencies, control, financing, institutions and ecosystems (Block et al. 2021; Cram et al. 2016; Hoegen et al. 2018; Nambisan 2017; Sussan and Acs 2017; Veit et al. 2014; von Briel et al. 2021). As lack of conceptual clarity and unclear boundary conditions are common challenges faced by digital entrepreneurship scholars, our chosen set of articles are all conceptual. Two literature reviews aim to extend theory (Kreuzer et al. 2022) and explore the roots of digital entrepreneurship to problematize the field and offer future research directions (Kollmann et al. 2022). Two papers report on case studies that were conducted to explore the importance of context in digital entrepreneurship (Keller et al. 2022) and build theory to explain post-launch product development in digital ventures (Lehmann and Recker 2022). The final paper develops a taxonomy from a case base of 100 start-ups that illustrates different ways AI can change business models (Weber et al. 2022).

**Table 1:** Digital Entrepreneurship (DE) articles of the special issue

Title	Link to entrepreneurship	Digital phenomenon	Theory	Method	Contribution
Eras of Digital Entrepreneurship – Connecting the Past, Present, and Future	Reviewing the broader entrepreneurship literature from early 90 s starting with the emergence of Internet technology as the first relevant enabler of digital venture creation	The paper studies how different digital phenomena (Internet technology, social media, cloud computing, blockchain) have been treated in the literature	The paper does not have a theory contribution. It is descriptive and tries to understand how the phenomenon of digital entrepreneurship is treated in the literature over time	Scoping literature review and technique of problematization to understand the roots and historical development of DE research	Contributes to research at the intersection between entrepreneurship and information systems literature by providing new insights into the eras of digital entrepreneurship from the past to the present and into the future
Pathways to Developing Digital Capabilities within Entrepreneurial Initiatives in Pre-Digital Organizations	Explores entrepreneurial initiatives (EIs) in pre-digital organizations (PDOs) to conceptualize digital intrapreneurship	Use of digital technologies to create new capabilities and explain how digital capabilities enable the creation of new products, services, and business models	Applies organizational identity theory to contribute to research on the relationship between IT and organizational identify	Single case study FoodLtd	Identify different pathways for developing digital capabilities and explain how managing a portfolio of pathways can enable digital transformation
The Effects of Digital Technology on Opportunity Recognition	Expand the impact of digital technology on a stage of the entrepreneurial lifecycle, specifically opportunity recognition	The paper explores how digital technology can enable opportunity recognition under conditions such as dispersed agency and blurred boundaries	Results extend opportunity recognition theory by illuminating the influence of digital technology	Literature review, case study and validation interviews	Introduce a new conceptual model that differentiates direct and transitive effects of digital technology on opportunity recognition
Offerings that are “Ever-in-the-Making” – How Digital Ventures Continuously DevelopTheir Products After Launch	Concepts such as effectuation, lean start-up and business modelling	Design/development of (digital) products/offerings by digital ventures. Adaptation of these offerings when new market/customer information becomes available and the product is already in the market	The paper does not apply a specific theory but leverages literature on product development in digital ventures	Multi-case study, grounded theory, inductive	Identification and description of three designing mechanisms that explain continuous post-launch product development in digital ventures: deploying complementary digital objects, architectural amplification, and porting
AI Startup Business Models – Key Characteristics and Directions for Entrepreneurship Research	Business model types of AI startups, differences of AI startup business models to traditional IT-related business models	The influence of AI on entrepreneurial activity	No specific theory applied, builds on literature related to business models and value creation logic	Taxonomy development approach of Nickerson et al. (2013)	A taxonomy of AI business model characteristic and archetypes. Discussion of differences that AI brings to business models and identifies future research directions

The entrepreneurship phenomena studied range from intrapreneurship within incumbent firms, to start-ups, to studying a specific stage (i.e., opportunity recognition) in the entrepreneurial process. Papers explore topics such as dispersed agency, blurred boundaries, digital capabilities, digital product development, and the evolution from Internet entrepreneurship to digital entrepreneurship and stages in between. One paper is atheoretical (Kollmann et al. 2022), two apply existing theory (Keller et al. 2022; Kreuzer et al. 2022), and two aim to build new theory by developing a taxonomy of AI business models (Weber et al. 2022) and a conceptual model of continuous post-launch product development (Lehmann and Recker 2022). Now that the general trends have been described, a brief summary of each article is provided.

Kollmann et al.’s ‘Eras of Digital Entrepreneurship: Connecting the Past, Present, and Future’ conducts a scoping literature review combined with the technique of problematization to understand the roots and historical development of digital entrepreneurship research. Its focus is on how different digital phenomena such as Internet technology, social media, cloud computing, and blockchain have been covered and treated in the entrepreneurship literature. The result is a timeline displaying the history of digital entrepreneurship (research) going back to the early 90’s. It thereby contributes to an understanding how digital entrepreneurship as a research field has emerged in parallel to the diffusion of digital technologies and important practical events.

Drawing on insights from a single case study, Keller et al. establish ‘Pathways to Developing Digital Capabilities within Entrepreneurial Initiatives in Pre-Digital Organizations’. One may deem this paper to be more aligned to digital transformation; however, the four pathways for developing digital capabilities are helpful in examining how new business models eradicate existing business models. Authors link their results to expand theory on digital capability development and provide practitioners with guidance on when using each pathway is appropriate.

Kreuzer et al.’s ‘The Effects of Digital Technology on Opportunity Recognition’ draws on insights from a comprehensive literature review validated by real-world case studies to explain how and why digital technologies alter the way organizations identify new opportunities. Their findings contribute to earlier research that investigated the nature of entrepreneurship enabled by digital technology (von Briel et al. 2021) by examining digital phenomena through the lens of opportunity recognition theory. The result of their study is a new theoretical model that calls attention to the relationship between digital technology and opportunity recognition. Specifically, their model differentiates two effects (direct and transitive) and evidence is provided to demonstrate how effects differ based on dispersed agency and blurred boundary conditions as underlying digital phenomena.

Recker and Lehmann’s ‘Offerings that are “Ever-in-the-Making”: How Digital Ventures Continuously Develop’ uses an inductive multi-case study to understand how digital technologies change entrepreneurial processes and outcomes. They identify and describe three design mechanisms (deploying complementary digital objects, architectural amplification, and porting) how digital ventures adapt their offerings when new information about customers and/or markets becomes available. The study connects to the wider business modelling literature and to popular entrepreneurship concepts such as effectuation and the lean start-up method.

Using a taxonomy development method, Weber et al. provide a classification of AI start-ups in their paper ‘AI Start-Up Business Models – Key Characteristics and Directions for Entrepreneurship Research’. They thereby relate to the literature stream on data-driven business models and contribute four different patterns of AI start-ups’ business models. First, the “AI-charged Product/Service Providers” offer services or products that have readily trained AI models embedded and can run without much configuration, such as packaged software for detecting prohibited items at airports. Second, the “AI Development Facilitators” provide development kits and interfaces to their customers for the simple specification and implementation of customized AI solutions with little IT expertise (e.g., build-your-own-chatbot). Third, the “Data Analytics Providers” help their customers in leveraging large internal and external data sources for better decision making (e.g., maintenance decisions for machines). Fourth, the “Deep Tech Researchers” drive the development of fundamental AI models and algorithms, which can be used by their customers to develop AI solutions using them. Based on these insights, the authors then discuss how AI-driven business models are different from traditional IT-related business models. They argue that AI capabilities allow new types of value creation by solving different customer pain points, that data can thereby play new and different roles, and that AI can bring an overall change in business logic through integrating complex mechanisms.

## Moving the Research Agenda Forward

### What has Not Changed?

Despite the many changes described above, some characteristics of entrepreneurship remain stable. Digital or not, the process of entrepreneurship is about the existence, discovery, and exploitation of entrepreneurial opportunities (Shane and Venkataraman 2000). While the emergence, forms and natures of entrepreneurial opportunities, as well as its mode and speed of exploitation, may have changed fundamentally in digital times, the basic process of opportunity discovery remains similar in digital or non-digital entrepreneurship. An entrepreneurial opportunity needs to be recognized as such. That is, an individual needs to learn about its existence and attach economic and/or social value to it. Only a subset of the population has the characteristics needed to discover such opportunities. Entrepreneurship research suggests two broad factors influencing the probability of opportunity discovery, namely prior information needed for opportunity identification (collected through various forms of education and experience) (Shane 2000) and cognitive or psychological properties necessary for opportunity valuation (Gielnik et al. 2014). Entrepreneurs differ from other individuals in these important aspects and this is true for digital and non-digital entrepreneurship alike.

Another aspect that has not changed in digital versus non-digital entrepreneurship is the decision to exploit opportunities. When deciding to exploit an opportunity, individuals weigh the benefits or value of the opportunity against the opportunity cost of doing something else. While the exact levels of value and cost may have changed in digital times, the tradeoff decision remains the same. As with opportunity discovery, the exploitation decision is highly subjective and depends on the characteristics of individuals in terms of his or her personality, access to information, and socio-economic situation. Finally, in both digital and non-digital entrepreneurship, there exists a distinction between innovative Schumpeterian and incremental Kirznerian forms of entrepreneurship. (Digital) ventures can be disruptive and through the exploitation of innovative opportunities, destroy existing market equilibria. However, they can also be incremental and exploit Kirznerian-type arbitrage opportunities. This important taxonomy has not changed in digital times. Most likely, the characteristics of individuals pursuing either form of opportunity also remain unchanged. Schumpeter (1934) describes entrepreneurs as visionary, optimistic, uncertainty tolerant, rational, confident, self-centered and motivated by power and need for achievement. We do not see a reason to believe that these entrepreneurial characteristics required for innovative Schumpeterian entrepreneurship have changed in digital versus non-digital entrepreneurship. The way towards disruption through innovation may have changed due to digitalization, but the fundamental characteristics and motivations of the entrepreneur remain the same. Entrepreneurs who do not possess these characteristics are, in Schumpeter’s view, not entrepreneurs but managers solving well-defined problems through planning. Such entrepreneurs or managers exist in both digital and non-digital ventures.

### What has Changed and Provides Future Research Opportunities?

As outlined in our theory section above, the digitalization and digital entrepreneurship has indeed changed some important taken-for-granted assumptions of entrepreneurship research and practice. As entrepreneurship as a phenomenon and academic discipline is highly interdisciplinary, these changes spill over to neighboring disciplines on the *individual* (e.g., psychology, well-being and health), *firm* (e.g., strategy, marketing and finance), *industry* (e.g., industrial organization), and *macro* levels (e.g., policy, economics, sociology). We will now briefly outline exemplary research questions derived from the novel aspects of digital entrepreneurship described in our theory section. Table 2 below provides a summary.

**Table 2:** A research agenda for digital entrepreneurship

Novel aspect	Exemplary research questions derived from the novel aspect
*New entrepreneurship actors*	
Digital platforms as a new start-up type	Which markets and industries are disrupted by digital platforms? How and when does disruption take place? What makes digital platforms successful? What are the financing, growth, and survival patterns? Who becomes a platform entrepreneur and what entrepreneurial and management skills are needed to establish and grow digital platforms?
Emergence of the gig economy	Who becomes active as entrepreneurs in the gig economy and what makes them successful? What are the effects of the gig economy on entrepreneur working conditions, well-being, and (mental) health?
*Changes to development and innovation processes*	
New digital product management lifecycles	How will the traditional new product development lifecycle change in the presence of digital technologies? What is the role of causality and temporality in entrepreneurial activities? What is the impact of digital technology on proximal milestones, or approximate outcomes? How and when do entrepreneurs use different types of capabilities (IT vs. digital)?
What’s new about ‘ever-in-the-making’ digital products?	How is value creation organized and entrepreneurial activities orchestrated when launching a digital product or service that is continuously evolving? What are the conceptual parameters and boundaries conditions that make ‘ever-in-the-making’ digital products unique? How does ‘ever-in-the-making’ differ from other related IT constructs (e.g., IT flexibility, enhancements)?
*New technologies and business models*	
Changes to the value proposition and product dimension of the business model	How can digital start-ups successfully balance their need for strong data lakes to create valuable products on the one hand and questions of data privacy (regulatory differences, ethics) on the other hand? Can approaches such as the lean start-up or design thinking help tackle such questions? How do differing regulatory settings of regions foster or hinder different types of digital business models or different types of AI start-ups (as provided by Weber et al. 2022) to emerge and thereby foster or hinder digital start-up ecosystems?
Changes to value architecture and network dimension of the business model	What are the strategies of start-ups to gain data access or gather large training sets given their liabilities of newness and smallness? How can they steer and leverage partnerships (e.g., with cloud or crowdsourcing providers) to gain access to such external resources? Can the digital enablement of such external resources theoretically challenge the concept of liabilities of newness and smallness for new ventures?
Changes to the value finance dimension of the business model	How can start-ups develop capabilities that are needed to work in dispersed teams on digital offers that are ‘ever-in-the-making’? What new types of mindset and skillsets are needed? What new leadership capabilities are required by founders? How can tensions between the need to monetize quickly (due to scarce resources) and the requirement (induced by network externalities) to keep digital services free be managed by start-ups? How and in which contexts can monetization still work without introducing negative path dependencies? What are the impacts of completely digitalized infrastructure, data capabilities, and data access strategies on start-up valuation by investors? How do new digital technologies change investment processes and shift investor-start-up relationships and power structures (for example via ICOs)?
*Policy and regulation*	
Digital policy as entrepreneurship policy and vice versa	Which digital policy concepts and instruments promote or hinder digital entrepreneurship? How does entrepreneurship policy interact with digital policy? How do differing privacy regulations impact the entrepreneurial development of regions?
Access to big data and AI	How can start-ups be granted access to big data so that they develop AI-based business models? How can and/or should intellectual property rights protect AI-based innovations?

*New Entrepreneurship Actors:* As outlined above, the digitalization and the digital economy have led to the emergence of new players and actors on the supply and demand side as well as new intermediaries. In particular digital platforms are an interesting and powerful new actor as a new form of intermediary. Many digital start-ups have platform business models and digital platforms represent a high share of successful IPOs in recent years. Future research should be concerned with how the particularities of digital platforms change our knowledge about how, when, and in which markets start-ups can disrupt existing industries and markets? This research question is in fact an old one that sites at the intersection of strategy, entrepreneurship, innovation, and industrial organization research. It goes back to Schumpeter (1934) and his notion of entrepreneurship as creative destruction of existing market equilibria. Schumpeter developed his concept of innovative entrepreneurship in the historical context of industrialization. However, the digitalization and digital platforms have changed the rules of the game and some of his notions about entrepreneurship and innovation may require an update. Hence, future research needs to re-examine what makes digital ventures (platforms) successful? What are their financing, growth, and survival patterns? What particular entrepreneurial and management skills are (no longer) needed? In some B2C markets, digital platforms have created a gig or sharing economy where individuals acting as (solo) entrepreneurs offer their services through a platform. While this gives them a wide reach, this can also create a strong (path) dependency. The gig economy as a phenomenon leads to many new research questions at the intersection of information systems, entrepreneurship, psychology, labor economics, and industrial sociology. Exemplary research questions would be: what are the de-facto and desired socio-demographic and human capital characteristics of gig entrepreneurs? What form of entrepreneurship is chosen by such entrepreneurs, e.g., hybrid, full-time or portfolio entrepreneurship? What are the consequences of gig entrepreneurship on the entrepreneur’s working conditions, well-being, and (mental) health?

*New Technologies and Business Models:* Creating and capturing value is central to the successful development of new business models. Focusing on *changes to the value proposition and product dimension* of business models, the homogenization (Yoo et al. 2010) has thereby fostered the crucial role of data as a central element of value creation. This holds for many new digital business models, particularly when looking at AI start-ups as argued in one of the papers of this Special Issue (Weber et al. 2022). However, given their liabilities of newness and smallness (Stinchcombe 1965), start-ups do not always have access to required data or need to gather data from their users for value creation. This can be a challenging task for new ventures given their liabilities of newness and smallness (Stinchcombe 1965) and ever-growing privacy regulation (e.g., GDPR). Hence, it might be worthwhile for future research to look into related questions such as: How can digital start-ups successfully balance their need for strong data lakes to create valuable products on the one hand and questions of data privacy (regulatory differences, ethics) on the other hand? Can approaches such as the lean start-up or design thinking help to tackle such challenges? Departing from the firm level, it might also be interesting to look into questions related to: how regulatory differences of regions allow for different types of business models? How do different types of AI start-ups (as provided by Weber et al. 2022) emerge? How regulatory differences foster or hinder digital start-up ecosystems?

As “data and users are the new oil” when creating value from digital technologies (The Economist 2017), start-ups also face hurdles in the *value architecture and network dimensions* of their business models. They need capabilities to develop large user bases and related data lakes themselves, or they need to build on top (i.e., piggyback) of the user bases or data sources of external partners (Parker et al. 2016; Stummer et al. 2018). Hence, the following questions could be of interest in future research: What are the strategies of start-ups to gain data access or gather large training sets given their liabilities? How can they steer and leverage partnerships (e.g., with cloud or crowdsourcing providers) to gain access to such external resources? Can the digital enablement of such external resources theoretically challenge the concept of liabilities of newness and smallness (Stinchcombe 1965) for new ventures? Moreover, scarcity of labor and high labor costs drive new ventures to make compromises when hiring talent. Giving additional freedom, such as the free choice of work location (i.e., working from home) and hiring offshore, can alleviate this challenge (Bradel et al. 2019). At the same time, such dispersed settings and high communicative needs due to continuous deployment can introduce additional challenges in management and leadership. It might therefore be of interest for future research to elaborate on questions such as: How can start-ups develop capabilities that are needed to work in dispersed teams on digital offers that are ‘ever-in-the-making’? What new types of mindset and skillsets are needed? What new leadership capabilities are required by founders?

Scarce resources and the inherent characteristics of digital technologies also dominate challenges in the *value finance dimension* of start-ups’ business models. Digital new ventures mostly require funding to develop needed capabilities and grow. There are two main ways to address this challenge, raise money internally via quick monetization or acquire external funding (e.g., via business angels or crowdfunding). However, going for quick monetization can introduce negative path dependencies, particularly in markets that exhibit network externalities (Steininger 2016). Venture capital can be hard to obtain over longer periods that are sometimes needed to establish a large user base. Given that many digital start-ups face network externalities, it might therefore be interesting to explore how, and in which contexts, monetization can still work without introducing negative path dependencies? Questions such as the following can thereby be of interest: How can tensions between the need to monetize quickly (due to scarce resources) and the requirement (induced by network externalities) to offer free digital services be managed by start-ups? What are the impacts of completely digitalized infrastructure, data capabilities, and data access strategies when it comes to start-up valuation by external investors? For the option to acquire funding externally, digitalization has also induced changes to the processes and power structures. While investors nowadays expect much closer insights into the current development stage and decisions via digital tools (Steininger 2022), start-ups also gain power due to social media coverage and hyped ways of external funding such as ICOs. An interesting question to explore might therefore be: How do new digital technologies change investment processes and shift investor-start-up relationships and power structures (for example via ICOs)?

*New product development process:* The process for managing the lifecycle of digital products has important differences that make it unique. For decades, researchers have assumed activities within the new product development process are causally related. While effectuation theory has gained prevalence in the entrepreneurship field (Sarasvathy 2001), we know relatively little about when and why entrepreneurs employ effectuation versus causation and how the role of digital technology influences entrepreneurial behaviors. As such, the following questions may guide future research opportunities: How does digital product management relate to existing disciplines such as design thinking and IT project management? What are the similarities and differences between IT capabilities and digital capabilities? Temporality is also interesting as rapid lifecycles were experienced during COVID-19 that jolted companies and industries to challenge institutional logics and experience new ways of organizing (Oborn et al. 2021). As such, future research might explore how entrepreneurial activities unfold over time in the presence (and absence) of digital capabilities? Finally, although ‘ever-in-the-making’ products leverage novel characteristics of digital technology, the idea that digital artifacts are incomplete and perpetually under development is not entirely new. As such, it is important future research consider how this concept relates to existing concepts such as flexible IT (Byrd and Turner 2000) and modifications such as personalization and customization.

*Policy and Regulation:* There are several opportunities for fruitful research regarding digital entrepreneurship policy. In particular, we lack micro-econometric evidence about the impact of policy measures on stimulating creation and growth of digital ventures. While venture capital firms, business angels, digital start-ups and other stakeholders of the digital eco-system constantly lobby for direct and indirect government support through subsidies and infrastructure investments, it remains unclear what concrete programs and policy measures produce the greatest effects. Some of them may actually be a waste of taxpayer’s money and crowd-out private investments and initiatives. Governmental digital hubs are becoming commonplace and often compete directly with private hubs and incubators. Rigorous evaluation research following established procedures in labor and innovation economics is needed to disentangle selection and treatment effects of digital entrepreneurship policy programs. Next to this line of research, we need to know more about the interrelationships that exist between digital, entrepreneurship, and innovation policy. These three policy fields often share common goals and we need to know more about how they support or hinder one another. Another fruitful area of future research concerns the regulation issue. Regulators in the US, EU and China struggle to find the right way to regulate the large digital platform monopolies to foster innovation and entrepreneurship. Different regulation alternatives exist ranging from breaking up the monopolies, granting start-ups forced access to platform networks and data, and simply forbidding platforms to become active in certain markets or industries. Future research on the most effective competition policy for digital entrepreneurship is a huge research opportunity with high theoretical and practical relevance.

To sum up, digital entrepreneurship is a fascinating research area that is evolving because it sits at the intersection of many disciplines. This topic of study has high practical and theoretical relevance as it questions some (but not all) of the core assumptions of entrepreneurship. We hope that our Editorial and the articles in the Special Issue inspire more research to better understand this important phenomenon.
